# Biochemical and Genetic Interactions of Phospholipase D Alpha 1 and Mitogen-Activated Protein Kinase 3 Affect Arabidopsis Stress Response

**DOI:** 10.3389/fpls.2019.00275

**Published:** 2019-03-18

**Authors:** Pavol Vadovič, Olga Šamajová, Tomáš Takáč, Dominik Novák, Veronika Zapletalová, Jean Colcombet, Jozef Šamaj

**Affiliations:** ^1^Department of Cell Biology, Centre of the Region Haná for Biotechnological and Agricultural Research, Faculty of Science, Palacký University Olomouc, Olomouc, Czechia; ^2^Institute of Plant Sciences Paris-Saclay, CNRS, INRA, Université Paris-Sud, Université d’Evry, Université Paris Diderot, Sorbonne Paris Cité, Université Paris Saclay, Orsay, France

**Keywords:** abscisic acid, *Arabidopsis thaliana*, genetic interaction, localization, mitogen-activated protein kinase 3, phospholipase D alpha 1, protein interaction, salt stress

## Abstract

Phospholipase D alpha 1 (PLDα1, AT3G15730) and mitogen-activated protein kinases (MAPKs) participate on signaling-dependent events in plants. MAPKs are able to phosphorylate a wide range of substrates putatively including PLDs. Here we have focused on functional regulations of PLDα1 by interactions with MAPKs, their co-localization and impact on salt stress and abscisic acid (ABA) tolerance in Arabidopsis. Yeast two-hybrid and bimolecular fluorescent assays showed that PLDα1 interacts with MPK3. Immunoblotting analyses likewise confirmed connection between both these enzymes. Subcellularly we co-localized PLDα1 with MPK3 in the cortical cytoplasm close to the plasma membrane and in cytoplasmic strands. Moreover, genetic interaction studies revealed that *pldα1mpk3* double mutant was resistant to a higher salinity and showed a higher tolerance to ABA during germination in comparison to single mutants and wild type. Thus, this study revealed importance of new biochemical and genetic interactions between PLDα1 and MPK3 for Arabidopsis stress (salt and ABA) response.

## Introduction

Phospholipases D (PLDs) represent enzymes that catalyze hydrolysis of phospholipids by producing phosphatidic acid (PA) and respective head group. In Arabidopsis PLDs are widespread family which include 12 isoforms distributed into six categories (α-, β-, γ-, δ-, ε-, and ζ), built on protein structure homology and biochemical properties of particular members (Qin and Wang, 2002; Bargmann and Munnik, 2006; Hong et al., 2016). PLDα1 is the most abundant member of the PLD family (Fan et al., 1999). PLDs has been reported to be involved in signaling events occurring in response to a multitude of stimuli, e.g., freezing, wounding, plant-pathogen interactions, dehydration, and salt stress (Wang, 2005; Bargmann and Munnik, 2006; Hong et al., 2010; Zhao, 2015). Earlier studies showed that PLDα1-derived PA plays a positive role in abscisic acid (ABA) mediated stomatal closure (Zhang et al., 2004; Jiang et al., 2014). PLDα1 also directly interacts with the Gα subunit of heterotrimeric G protein, to inhibit stomatal opening (Zhao and Wang, 2004; Mishra et al., 2006). In addition, PLDα1 is required for ethylene- and ABA-regulated leaf senescence (Fan et al., 1997) and PLDα1-derived PA inhibits CTR1 kinase activity, thus promotes ethylene response and activates mitogen-activated protein kinase (MAPK) cascade (Testerink et al., 2007; Li et al., 2009).

Mitogen-activated protein kinase signaling pathways are linked multi-enzymatic complexes, whose members are phosphorylated sequentially, thereby transducing signals within the cell (Šamajová et al., 2013a,b; Komis et al., 2018). In all eukaryotes they are regularly organized into conserved three-tiered modules composed of MAPK kinase kinase (MAPKKK), MAPK kinase (MAPKK), and MAPK. MAPKs, as the final kinases in the cascade, phosphorylate substrates which regulate diverse cellular and physiological processes (Lee et al., 2015). In Arabidopsis genome, approximately 110 genes of MAPK module components were identified: 60–80 genes for MAPKKK, 10 genes for MAPKK and 20 genes for MAPK (Jonak et al., 2002; MAPK Group, 2002; Dóczi et al., 2012).

Mitogen-activated protein kinases play an important role in plant adaptation and are involved in plant responses to hormones, biotic and abiotic stress (Colcombet and Hirt, 2008; Rodriguez et al., 2010; Sinha et al., 2011; Liu, 2012; Šamajová et al., 2013a; Smékalová et al., 2014; Latrasse et al., 2017). Signaling by MAPK pathway is also involved in the regulation of fundamental physiological processes such as cell division, growth, development, differentiation, cytoskeletal reorganization or programmed cell death (Takahashi et al., 2004; Franklin-Tong and Gourlay, 2008; Müller et al., 2010; Beck et al., 2011; Šamajová et al., 2013b; Komis et al., 2018).

Mitogen-activated protein kinase signaling specificity, amplitude and duration is regulated at several levels. These include the assembly and composition of a given MAPK module (Colcombet and Hirt, 2008), deactivation of single or multiple elements of a cascade by phosphatases (Meskiene et al., 1998, 2003; Ulm et al., 2002; Luan, 2003; Naoi and Hashimoto, 2004), and by specific spatial organization and subcellular localization of a particular MAPK module (Komis et al., 2011, 2018; Šamajová et al., 2013b).

Salt stress is considered as one of the major limiting factors in crop production. High salinity is a result of high concentrations of sodium and chloride ions in the soil resulting in hyperosmotic conditions which hinder the water and nutrients uptake by plants (Ismail et al., 2014; Selvakumar et al., 2014). So far, two MAPK pathways MEKK1-MKK2-MPK4/MPK6 and MKK9-MPK3/MPK6 were identified to transduce salt stress triggered signaling in Arabidopsis (Teige et al., 2004; Xu et al., 2008). The latter one is also involved in ethylene and camalexin biosynthesis (Xu et al., 2008; Yoo et al., 2008). Previously, *mpk3* mutant showed sensitivity to higher salinity conditions (Persak and Pitzschke, 2013; Pitzschke et al., 2014). On the other hand, *mekk1* together with *mkk9* mutants are more tolerant to the salt (Su et al., 2007; Yoo et al., 2008).

Interestingly, PLDα1 and PA appear as important regulators of MAPK signaling. First, PLD-derived PA activates MAPK in response to wounding in soybean leaves (Lee et al., 2001). Second, PA produced by PLDα1 during salt stress, binds to MPK6 and stimulates its kinase activity, which in turn phosphorylates and activates SOS1 representing Na^+^/H^+^ antiporter (Yu et al., 2010). Coherently, *pldα1* mutant which produces less PA, shows decreased MPK6 activity, higher intracellular accumulation of Na^+^ ions in leaves and higher susceptibility to salinity. On the other hand, MAPKs might regulate PLDα1, because this protein was overabundant in *mpk6* mutant in a comparative proteomic study (Takáč et al., 2016). PLDα1 was also predicted as a promising interaction and phosphorylation target of MAPKs, because it possesses a MAPK-specific phosphorylation site S481 as well as MAPK specific docking site in its amino acid sequence (Takáč et al., 2016). This protein is phosphorylated during drought stress and ABA response in Arabidopsis at this particular phosphorylation site (Umezawa et al., 2013).

In this study, we further explored biochemical and genetic interactions of PLDα1 and MPK3, and investigated their relevance for plant resistance to salt and response to ABA.

## Materials and Methods

### Plant Material, Mutant Screens, Characterization and Generation of Double Mutants

Seedlings were grown vertically on half-strength MS media (Murashige and Skoog, 1962) supplemented with 0.6% (w/v) gellan gum for 14 days in controlled environmental conditions with 21°C and a 16 h/8 h (light/dark) photoperiod. The illumination intensity was 150 μmol.m^-2^.s^-1^. Twelve to fifteen days old plants were transferred to soil and cultivated in growth chamber in controlled environmental conditions as specified above.

*Arabidopsis thaliana* ecotype Columbia-0 (Col-0) was used as the background in all mutant plants in this study. We have used T-DNA insertion line *pldα1-2* (SALK_53785; Zhang et al., 2004) and the T-DNA insertion line *mpk3-1* (SALK_151594; Wang et al., 2007).

The primers to check the T-DNA insertions in all Salk lines were designed according to the SIGnAL iSect Tool^1^, and polymerase chain reaction (PCR) was performed using genomic DNA from seedlings. The double homozygous mutants in *PLDα1* and *MPK3* genes used in this study were identified by PCR genotyping in the second generation of the progeny of a cross between single mutants.

To generate Gateway DONOR clones (pDONR) containing *PLDα1* open reading frame (At3g15730), a PCR fragment was amplified from Col-0 cDNA using iProof enzyme (Bio-Rad) and primer pairs 5′-GGA GAT AGA ACC ATG GCG CAG CAT CTG TTG CAC-3′/5′-TCC ACC TCC GGA TCM CCT GCC TCC AAT CCT TAC AAC C-3′ and recombined in pDONR207 using Gateway technology according to the manufacturer protocol (Invitrogen). Clones with and without the stop codon (referred as pDONR-PLDα1-STP and pDONR-PLDα1-END) were selected, allowing N- and C-terminal protein fusions. Clones were systematically sequenced. For functional studies, *PLDα1* ORF was recombined using the LR enzyme mix following the manufacturer’s indications (Invitrogen^TM^) in devoted plasmids.

### Yeast Two-Hybrid Analysis

For yeast two-hybrid assays, *PLDα1* ORF was recombined from pDONR-PLDα1-STP vector in pDEST22 Gateway vector via LR reaction (Invitrogen^TM^). Both pDEST22-PLDα1 and pDEST32m-MPK3/4/6 (Berriri et al., 2012) were co-transformed in the yeast two-hybrid reporter strain MaV203 (Vidal et al., 1996) using a classical lithium/polyethylene glycol/heat shock method (Schiestl and Gietz, 1989). Transformed colonies were selected on medium lacking Leu and Trp [SC with 0.2% dropout-L-W (United States Biological) at 30°C for 2 days]. Single colonies were grown overnight in the same medium without agar and then diluted 200 times, and 5 μL were spotted on an SC plate [SC with 0.2% dropout-L-W-U (United States Biological)]. After 11 days of cultivation we scanned and measured yeast density as mean intensity value of yeast spots grown on SD-L-W-U medium and subtracted unspecific background of spots mean intensity of both empty vector combinations. For evaluation of specific interaction of PLDα1 with MPK3/4/6 we subtracted signals from yeast spots of empty pDEST22 and pDEST32m vectors from the signals of spots with relevant combinations of MPKs and PLDα1.

### Bimolecular Fluorescence Complementation Assay

*Phospholipase D alpha 1* ORF was recombined from pDONR-PLDα1-END vector in pBiFC2 and pBiFC4 Gateway vectors (Thermo Fischer Scientific) via LR reaction (Invitrogen^TM^), allowing the C- and N-terminal fusion of PLDα1 with the N- and C-terminal YFP moieties, respectively (Azimzadeh et al., 2008). *Agrobacterium tumefaciens* C58C1 strain containing vectors with *PLDα1*, *MPK3/4/6*, *MKK2* and empty vectors were used to co-infiltrate in *Nicotiana benthamiana* leaves as previously described (Sparkes et al., 2006). After 72 h, leaves of transformed plants were observed on spinning disk microscope (Cell Observer SD, Carl Zeiss, Germany) equipped with Plan-Neofluar 10×/0.30 (Carl Zeiss, Germany) objective. All samples were imaged with excitation laser 514 nm and with emission filter BP535/30 for YFP. Chlorophyll was imaged with excitation laser 561 nm and with emission filter BP629/62. Final figures showing representative images from two independent experiments were created from 25 Z-stacks (total thickness 93. 36 μm) flatten to one image with Orthogonal Projection function of ZEN 2010 (blue edition) software. Average of mean intensity values for YFP channels from two measurements were normalized according to chlorophyll while unspecific background signal of empty vectors was subtracted.

### Co-localization of mCherry-Tagged MPK3 With PLDα1-YFP

*MPK3:mCherry* and *mCherry:MPK3* constructs driven by 1485 bp long native promoter sequence and complete coding region of *MPK3* were prepared by MultiSite Gateway^®^ technology. This constructs were cloned in to the pB7m34GW binary vector. YEB medium (5 ml) including appropriate selection antibiotics was individually inoculated with *A. tumefaciens* GV3101 previously transformed with binary vectors coding PLDα1-YFP, MPK3-mCherry and mCherry-MPK3 proteins. Cultures grown at 28°C, 220 rpm to OD_600_ 0.4 were pelleted at 3000 g, 4°C for 10 min. Pellets were resuspended in 5 ml buffer including 10 mM MgCl_2_, 10 mM MES (pH 5.6) and 150 μM acetosyringone and subsequently incubated at room temperature for 2 h. Bacterial culture containing *MPK3* construct was mixed with culture carrying *PLDα1* construct using syringe. Such mixed cultures were infiltrated into 6 week old *N. benthamiana* leaves. After agroinfiltration, plants were covered for 24 h with transparent plastic bags and maintained in fytotron. After 48 h, co-transformed epidermal cells expressing both *PLDα1-YFP* and *mCherry-MPK3* constructs were observed on spinning disk microscope (Cell Observer SD, Carl Zeiss) equipped with EC Plan-Neofluar 40×/1.30 Oil DIC objective (Carl Zeiss, Germany). Cells were imaged with excitation laser 561 nm and with emission filter BP629/62 for mCherry, excitation laser 514 nm and with emission filter BP535/30 for YFP, excitation laser 639 nm and with emission filter BP690/50 for chlorophyll B.

### Cloning of *proMPK3::MPK3:YFP* Constructs and Stable Plant Transformation

Two different vectors were used for preparing of *proMPK3::MPK3:YFP* construct, pGREEN 0229 containing genomic sequence of *MPK3* gene with putative native promoter, and pGEMTeasy containing sequence of *YFP* gene. *MPK3* gene with promoter was cleaved from pGREEN 0229 vector by *BamH*I, whereas YFP gene by combination of *BamH*I and *Bgl*II restriction enzymes. Restriction was verified by agarose electrophoresis and the required segments of DNA were cut from gel, purified and ligated by T4 DNA ligase (Invitrogen) according to relevant protocol. Newly formed construct was transformed to *Escherichia coli* strain DH5α by heat shock at 42°C and transformation was checked by restriction analysis. Correctness of *proMPK3::MPK3:YFP* construct was verified by sequencing. This vector was subsequently used for transformation of *A. tumefaciens* strain GV3101 by heat shock at 37°C. *A. thaliana* Col-0 plants were transformed by floral dip method according to Clough and Bent (1998). Transformed plants were selected on BASTA.

### Whole Mount Immunofluorescence Labeling

Immunolocalization of microtubules, PLDα1 and MPK3 in root wholemounts was done as described previously (Šamajová et al., 2014). Samples were immunolabeled with rat anti-α-tubulin (clone YOL1/34; AbD Serotec), rabbit anti-phospholipase D alpha 1/2 (Agrisera, Sweden), rabbit anti-MPK3 (Sigma-Aldrich) primary antibodies diluted 1:300, 1:500, and 1:350, respectively in 3% (w/v) BSA in PBS at 4°C overnight. In the case of double co-immunolocalization a sequential immunolabeling was performed. Secondary antibodies including Alexa-Fluor 488 goat anti-rat and Alexa-Fluor 546 goat anti-mouse or Alexa-Fluor 488 goat anti-mouse and Alexa-Fluor 546 goat anti-rabbit IgGs were diluted 1:500 in PBS containing 3% (w/v) BSA for 3 h (1.5 h at 37°C and 1.5 h at room temperature). Where necessary, nuclei were counterstained with DAPI. Microscopic analysis of immunolabeled samples was performed with a Zeiss 710 Confocal Laser Scanning Microscope (CLSM) platform (Carl Zeiss, Jena, Germany), using excitation lines at 405, 488, and 561 nm from argon, HeNe, diode and diode pumped solid-state lasers. Images were processed using ZEN 2010 software (black edition), Photoshop 6.0/CS, and Microsoft PowerPoint.

### Immunoblotting Analysis

Roots and above ground parts of 14 days old Col-0, *pldα1-2* and *mpk3-1* single mutant and *pldα1-2mpk3-1* double mutant plants were homogenized in liquid nitrogen to fine powder and proteins were extracted in E buffer [50 mM HEPES (pH 7.5), 75 mM NaCl, 1 mM EGTA, 1 mM MgCl_2_, 1 mM NaF, 10% v/v glycerol], Complete^TM^ EDTA-free protease inhibitor and PhosSTOP^TM^ phosphatase inhibitor cocktails (both from Roche, Basel, Switzerland). Following centrifugation at 13000 *g* for 15 min at 4°C, supernatants were supplemented with 4 times concentrated SDS sample buffer [in final concentration 62.5 mM Tris–HCl (pH 6.8), 2% (w/v) SDS, 10% (v/v) glycerol, 300 mM 2-mercaptoethanol] and boiled at 95°C for 5 min. Proteins were separated by SDS–PAGE (MINI-Protean II cell system, Bio-Rad) on 10% gels. Identical protein concentrations were loaded for each sample. Proteins were transferred to polyvinylidene difluoride (PVDF) membranes (GE Healthcare) in a wet tank unit (Bio-Rad) overnight at 24 V using transfer buffer [25 mM Tris, 192 mM glycin, 10% (v/v) methanol].

For immuno-detection of proteins, the membranes were blocked in a mixture of 4% w/v low-fat dry milk and 4%w/v bovine serum albumin in Tween-20 supplemented Tris-buffered-saline (TBS, 100 mM Tris–HCl; 150 mM NaCl; pH 7.4, X% (v/v) Tween-20) at 4°C overnight. Afterward, membranes were incubated with anti-PLDα1/2 (Agrisera, Sweden; diluted 1:3000), anti-beta tubulin (Sigma-Aldrich, diluted 1:2000), anti-MPK3 (Sigma-Aldrich, diluted 1:3000), anti-MPK4 (Sigma-Aldrich, diluted 1:1000), anti-MPK6 (Sigma-Aldrich, diluted 1:15000) antibodies and polyclonal antibody against mammalian phosphorylated ERK1/2 [phospho-p44/42 (pERK); Cell Signaling; Danvers, ME, United States, diluted 1:1000]. Antibodies were diluted in TBS-T containing 1% w/v BSA and incubated at room temperature for 1.5 h (anti-PLDα1/2 and anti-beta tubulin) or overnight at 4°C. Following five washing steps in TBS-T, membranes were incubated with a horseradish peroxidase conjugated goat anti-rabbit IgG secondary antibody (Santa Cruz Biotechnology, Santa Cruz, CA, United States) and goat anti-mouse IgG secondary antibody (Santa Cruz Biotechnology, Santa Cruz, CA, United States), diluted 1:5000 in TBS-T containing 1% w/v BSA at room temperature for 1.5 h. After five washing steps in TBS-T, proteins were detected by incubating the membranes in Clarity Western ECL substrate (Bio-Rad, Hercules, CA, United States). Luminescence was detected using ChemiDoc MP documentation system (Bio-Rad). The band intensities were quantified using Image Lab Software (Bio-Rad) and the data were statistically evaluated using Student’s *t*-test. Immunoblot analyses were performed in three biological replicates.

### Salt Sensitivity Assay

To study roles of *PLDα1* and *MPK3* in survival of Arabidopsis seedlings under salt stress conditions we used 4 days old seedlings of wild type (Col-0), *pldα1-2* and *mpk3-1* single mutants and *pldα1-2mpk3-1* double mutants grown on half-strength MS media and transferred them on media supplemented with 150 mM NaCl. Together 48 seedlings from each genotype were monitored and the plant viability was determined as percentage of survival plants with green true leaves at 7, 14, and 21 days after transfer. Experiment was done in three biological replicates.

### Germination Assay Under Salt Stress Conditions and ABA Treatment

Seeds from wild type (Col-0), *pldα1-2* and *mpk3-1* single mutants and *pldα1-2mpk3-1* double mutant were sown on half-strength MS media containing 0, 100, 125, 150, and 175 mM NaCl or 0, 0.5, 1, 3, and 5 μM ABA, respectively. Plates were kept at 4°C for 48 h and transferred in growth chamber under standard conditions. Germination ratio (% of seeds with visible radicle to all examined seeds) was evaluated under stereomicroscope 24, 48, and 72 h after transfer to chamber. Experiment was repeated in three replicates.

## Results

### MPK3 Physically Interacts With PLDα1

**FIGURE 1:**
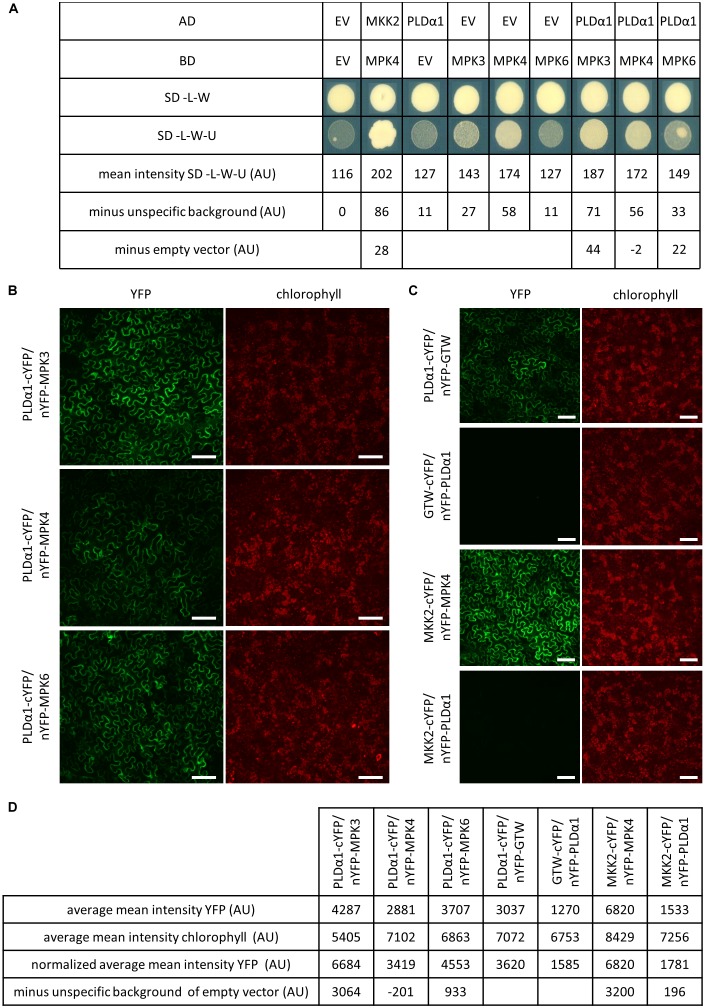
PLDα1 interacts with MPK3. **(A)** Yeast two-hybrid assay of PLDα1 and MPK3/4/6. *PLDα1* full-length cDNA was cloned and transferred into yeast two-hybrid Gateway^TM^ pDEST22 prey vector (Invitrogen^TM^) and *MPK3/4/6* were introduced into yeast two-hybrid Gateway^TM^ pDEST32m bait vector (Berriri et al., 2012) (Invitrogen^TM^). *PLDα1* as a GAL4-activating domain (AD) and *MPK3/4/6* as a GAL4-binding domain (BD) were co-transformed into yeast strain MaV 203. Interaction was monitored by measuring of yeast colonies growth intensity on selective plates (SC-L-W-U) and on control plates (SC-L-W). As a positive control was used *MKK2* as an AD and *MPK4* as a BD. Combinations of *PLDα1* and *MPK3/4/6* with corresponding empty vectors were considered as negative controls. Measurements of *PLDα1* and *MPK3/4/6* yeast densities as mean intensity of colonies grown on SD-L-W-U media subtracted from empty vector (EV) unspecific background obtained from colonies growth on SD-L-W-U and subtracted from corresponding *EV* and *MPK3/4/6* yeast colony densities combinations. The strongest yeast colony intensity was obtained with *PLDα1* and *MPK3* vector combination (44 AU). SC-L-W: synthetic dropout medium without leucine and tryptophan. SC-L-W-U: synthetic dropout medium without leucine, tryptophan and uracil. EV: empty vector (pDEST22 or pDEST32). **(B)** Bimolecular fluorescence complementation (BiFC) assay in *N. benthamiana* leaf pavement cells showing that PLDα1 strongly interacts with MPK3 and weakly interacts with MPK6 in plant cells. PLDα1 combined with MPK4 does not show any interaction after subtraction of unspecific background of empty vector. **(C)** Relevant controls for BiFC assay. MKK2 combined with MPK4 serve as a positive control. MKK2 combined with PLDα1 serve as a negative control while PLDα1 with empty vector GTW and empty vector GTW with PLDα1 serve for subtraction of unspecific background of empty vectors. **(D)** Measurements of average mean intensity value of YFP and chlorophyll, normalized average mean intensity of YFP according to chlorophyll and final intensity after subtraction of unspecific background of empty vectors. cYFP, C-terminal version of YFP; nYFP, N-terminal version of YFP; nYFP-GTW, empty vector pBIFC2; GTW-cYFP, empty vector pBIFC4; AU, arbitrary units. YFP fluorescence is shown in false green color. Scale bars = 100 μm.

Phospholipase D alpha 1 is suspected to be phosphorylated by stress activated MAPKs (Bargmann et al., 2009a). In order to investigate which of MPK3, MPK4, and MPK6 were able to interact with PLDα1, we carried out a pair-wise yeast two-hybrid assay (Figure 1A). PLDα1 fused to GAL4 activation domain was co-expressed in yeast with MPK3, MPK4, and MPK6 proteins fused to the GAL4 binding domain. Combination of *MKK2* and *MPK4* genes was used as a positive control for testing interaction while combination of both empty vectors (EV) was used for subtraction of unspecific background. Viability and growth of yeast colonies carrying *PLDα1*/*MPK4* and *PLDα1*/*MPK6* plasmid combinations on selective plates was inhibited and in the case of *PLDα1*/*MPK6* showed moderate growth (22 AU) after subtraction of single transformed EV with *EV*/*MPK6* combination. *EV*/*MPK4* plasmid combination showed some unspecific growth on selective plates (Figure 1A), and subtraction of this unspecific growth from *PLDα1*/*MPK4* combination resulted in negative mean intensity (-2 AU). However, colonies carrying *PLDα1*/*MPK3* plasmid combination exhibited strongest growth (44 AU) from all *PLDα1* and *MPKs* combinations even after subtraction of unspecific growth of *EV/MPK3* on selective plates (Figure 1A). These results indicated that PLDα1 is able to interact in yeast with MPK3 and weakly with MPK6 but likely not with MPK4.

To verify whether PLDα1 and MPK3 interact together also *in planta*, we performed bimolecular fluorescent complementation (BiFC) assay by using transient transformation of *N. benthamiana* leaves and spinning disc microscopy (Figure 1B,C). Measured picture mean intensity of YFP from two measurements was averaged and normalized according to chlorophyll intensity. Results showed strong normalized average mean intensity of YFP signal suggesting interaction of co-transformed PLDα1 and MPK3 (Figure 1B,D). Strong signal 3064 AU (after subtraction of unspecific background of empty vector PLDα1/GTW, Figure 1C,D) appeared near to the plasma membrane and around the nucleus of leaf epidermal cells. Much weaker normalized average mean intensity of YFP was observed (also after subtraction of unspecific background of empty vector PLDα1/GTW, Figure 1C) with PLDα1/MPK6 pair combination (933 AU, Figure 1D). Conversely PLDα1/MPK4 pair combination showed negative YFP signal (-201 AU, Figure 1D) after subtraction of unspecific background of empty vector in leaf epidermal cells. As a positive control, we have used a pair of interacting proteins, namely MKK2 and MPK4. MKK2 and PLDα1 protein pair was used as a negative control (Figure 1C). Based on the results of both interaction assays we conclude that PLDα1 physically interacts with MPK3 under *in vitro* (Figure 1A) and *in planta* (Figure 1B) conditions.

### Co-localization of PLDα1 and MPK3

**FIGURE 2:**
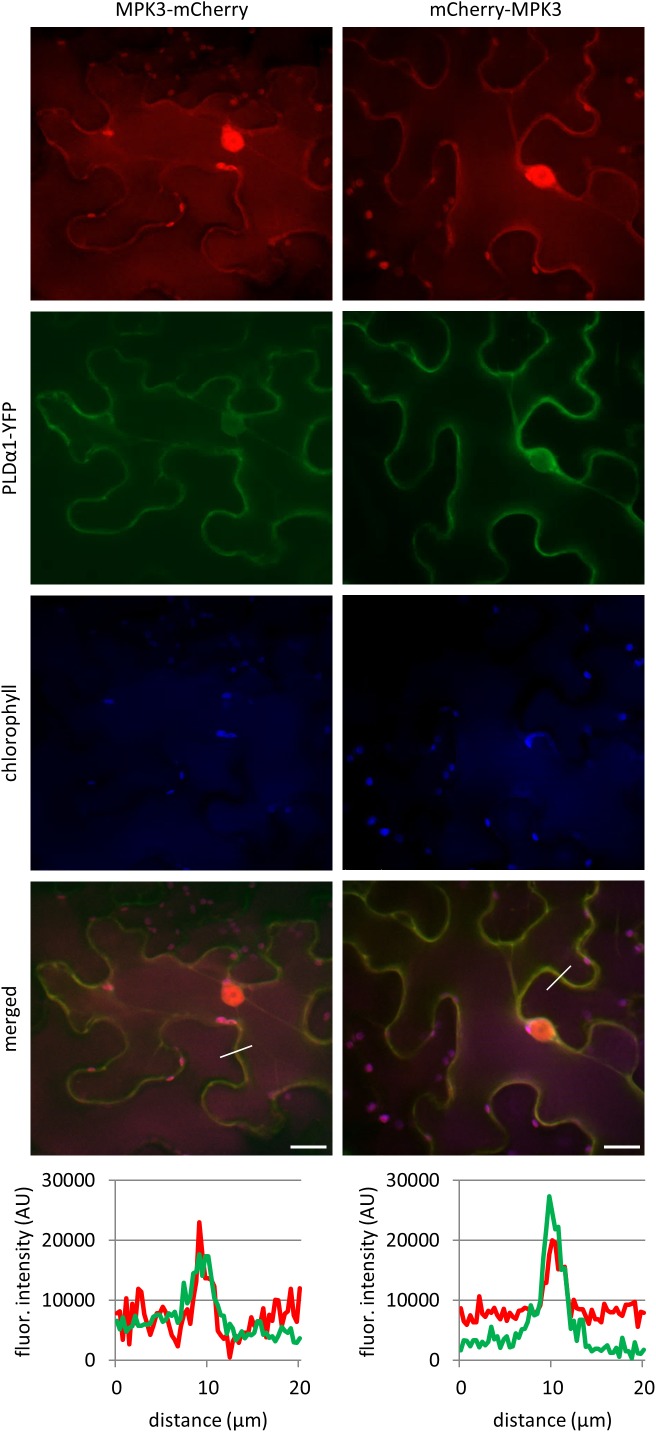
*In vivo* co-localization of MPK3-mCherry and mCherry-MPK3 with PLDα1-YFP. Co-localization of mCherry tagged MPK3 under the control of native *MPK3* promotor (in red) and YFP tagged PLDα1 driven by *PLDα1* own promoter (in green) both transiently co-expressed in *N. benthamiana* leaf pavement cells. Note co-localization of PLDα1-YFP with mCherry tagged MPK3 at cell peripheries close to the plasma membrane and in cytoplasmic strands. Profile intensity of fluorescence distribution of both MPK3 (red line) and PLDα1 (green line) based on distance are indicated by lines in merge pictures. Analysis was made on spinning disk microscope. Scale bar = 20 μm.

In order to test whether MPK3 co-localizes with PLDα1 *in planta* we transiently co-transformed leaves of *N. benthamiana* with *A. tumefaciens* containing combinations of constructs *PLDα1:YFP* with *MPK3:mCherry* and *PLDα1:YFP* with *mCherry:MPK3*. Both MPK3-mCherry and mCherry-MPK3 protein fusions were localized in nuclei, cytoplasmic strands and cortical cytoplasm close to the plasma membrane (Figure 2). Co-localization of PLDα1 with MPK3 has been documented similarly in the cortical cytoplasm close to the plasma membrane and in cytoplasmic strands (Figure 2). *In planta* co-localization of PLDα1 and MPK3 corroborated above-mentioned interaction studies (Figure 1) between these two proteins. Further studies on Arabidopsis lines stably expressing either YFP-tagged MPK3 (Supplementary Figure S1) or YFP-tagged PLDα1 (Novák et al., 2018), both under their native promoters, showed common localization to the vicinity of plasma membrane and cytoplasmic punctate structures. Additionally, MPK3-YFP was found in nuclei and nucleoli (Supplementary Figure S1).

**FIGURE 3:**
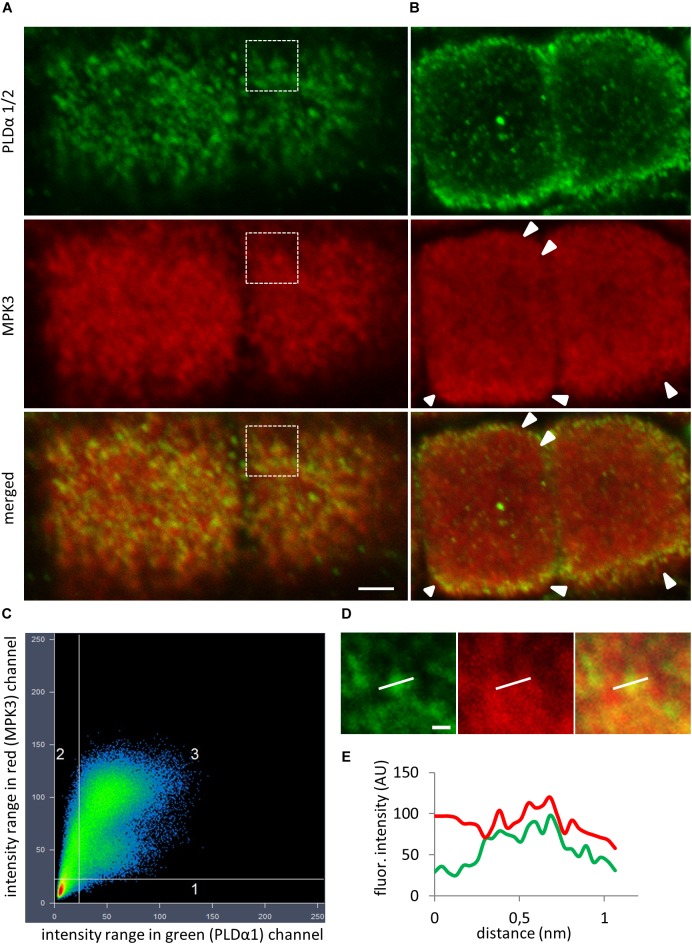
*In situ* co-immunolocalization of PLDα1/2 and MPK3 in fixed epidermal root cells of Col-0. **(A)** Immunofluorescence localization showed punctate labeling of PLDα1 and MPK3 with close association or partial co-localization in cortical cytoplasm close to the plasma membrane (PM) of epidermal root cells. **(B)** Localization of MPK3 close to the PM (arrowheads). **(C)** Scatter plot revealed positive colocalization (Pearson’s coefficient *R* = 0.88) between PLDα1 and MPK3 in a root epidermal cells shown in **(A)**. Horizontal axis represents green channel (PLDα1) and vertical axis represents red channel (MPK3). **(D)** Details corresponding to boxed area in **(A)** showing punctate labeling of PLDα1 and MPK3. **(E)** Profile intensities of fluorescence distribution of MPK3 (red line) and PLDα1 (green line) along white lines indicated in **(D)**. Scale bar = 2 μm.

To test whether the native PLDα1 and MPK3 also co-localize in Arabidopsis, we performed immunofluorescence labeling using anti-PLDα1/2 and anti-MPK3 antibodies (Figure 3A,B). The anti-PLDα1/2 recognizes both PLDα1 and PLDα2. Since PLDα2 is pollen specific, this antibody recognizes only PLDα1 in other plant organs. CLSM analysis on immunofluorescently labeled Arabidopsis root tips showed punctate labeling of both PLDα1 and MPK3 with close association or partial co-localization in the cortical cytoplasm close to the plasma membrane of epidermal root cells (Figure 3A). Quantitative analysis of fluorescence confirmed positive co-localization of PLDα1 and MPK3 (Pearson’s coefficient *R* = 0.88; Figure 3C–E). Additionally, MPK3 was localized close to the plasma membrane in root epidermal cells (Figure 3B). Further no differences in the distribution pattern of MPK3 as well as possible association with cortical microtubules was found in immunolabeled epidermal root cells of *pldα1-*2 mutant in comparison to the wild type control (Supplementary Figure S2).

### Abundance and Phosphorylation of MPK3 Requires the Presence of PLDα1 in Arabidopsis

**FIGURE 4:**
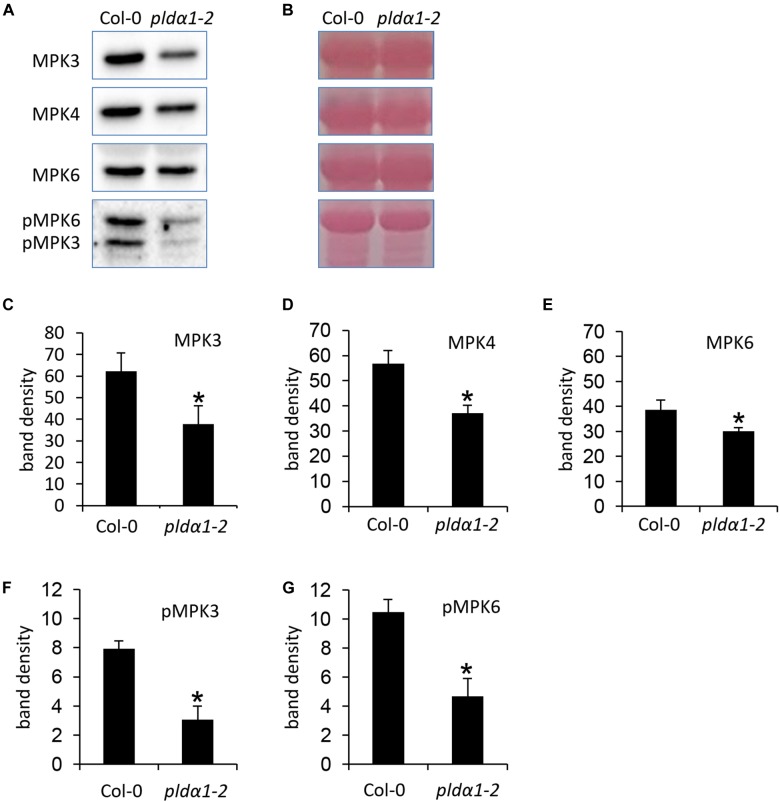
Abundance and activity of MAPK in above ground parts of *pldα1-2* mutant and wild type. **(A)** Immunoblots probed with anti-MPK3, anti-MPK6, anti-MPK4 antibodies and phosphospecific pERK 44/42 antibody recognizing phosphorylated MPK3 (pMPK3) and MPK6 (pMPK6). **(B)** Visualization of proteins transferred on PVDF membranes. **(C–G)** Quantification of band intensities obtained in **(A)**. Asterisks indicate significant differences between mutants and wild type at *p* < 0.05 according to Student’s *t*-test. Error bars represent SD.

It is known that MPK6 is activated by PLDα1-produced PA in response to the salt stress (Yu et al., 2010). To monitor the impact of PLDα1 deficiency on abundances of MPK3/4/6 and phosphorylation of MPK3 and MPK6, we performed an immunoblotting analysis using anti-MPK3, anti-MPK4, anti-MPK6 and anti-phospho-p44/42 MAPK (Erk1/2) antibodies in above ground parts of *pldα1-2* mutant. The anti-phospho-p44/42 MAPK (Erk1/2) antibody recognizes phosphorylated forms of MPK3 and MPK6 in Arabidopsis (Frei dit Frey et al., 2014; Takáč et al., 2016). We observed a significant decrease in abundances of MPK3, MPK4, and MPK6 in *pldα1-2* (Figure 4A–E). Phosphorylation of both MPK3 and MPK6 was substantially reduced in *pldα1-2* mutants as well (Figure 4A,B,F,G). Images of the entire immunoblots are presented in Supplementary Figures S3–S6. These results show that similarly to the previously published data on MPK6 (Yu et al., 2010), PLDα1 deficiency negatively affects the abundance and phosphorylation of MPK3.

### The *pldα1-2mpk3-1* Double Mutant Is More Tolerant to the Salt Stress

**FIGURE 5:**
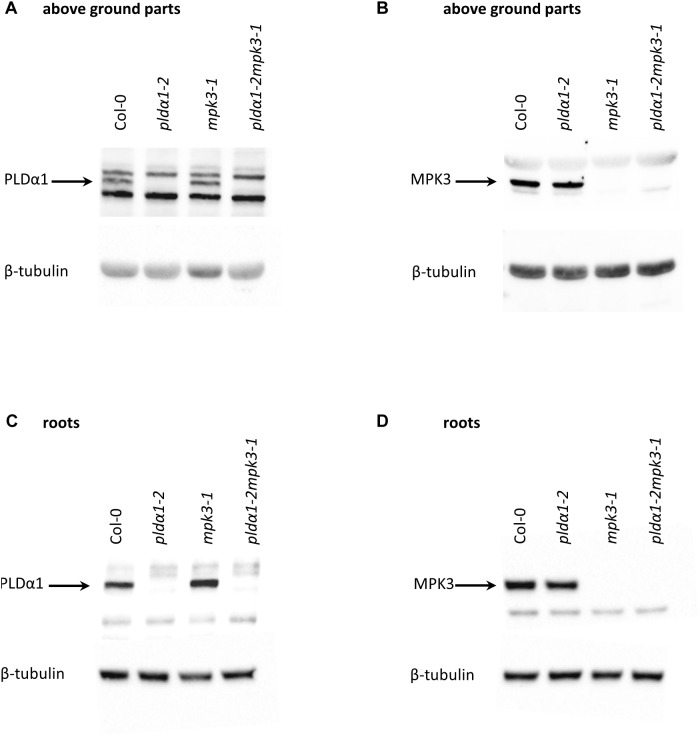
Immunoblotting analysis of *pldα1-2, mpk3-1* single mutant and *pldα1-2mpk3-1* double mutant plants (in F3 generation). Protein extracts were prepared from the above ground parts and roots of adult *pldα1-2mpk3-1* double mutant plants, from *pldα1-2, mpk3-1* mutant plants and Col-0 plants. Immunoblots were probed with **(A,C)** anti-PLDα1/2 and **(B,D)** anti-MPK3 antibodies. For loading control, membranes were probed with anti-β-tubulin antibody.

**FIGURE 6:**
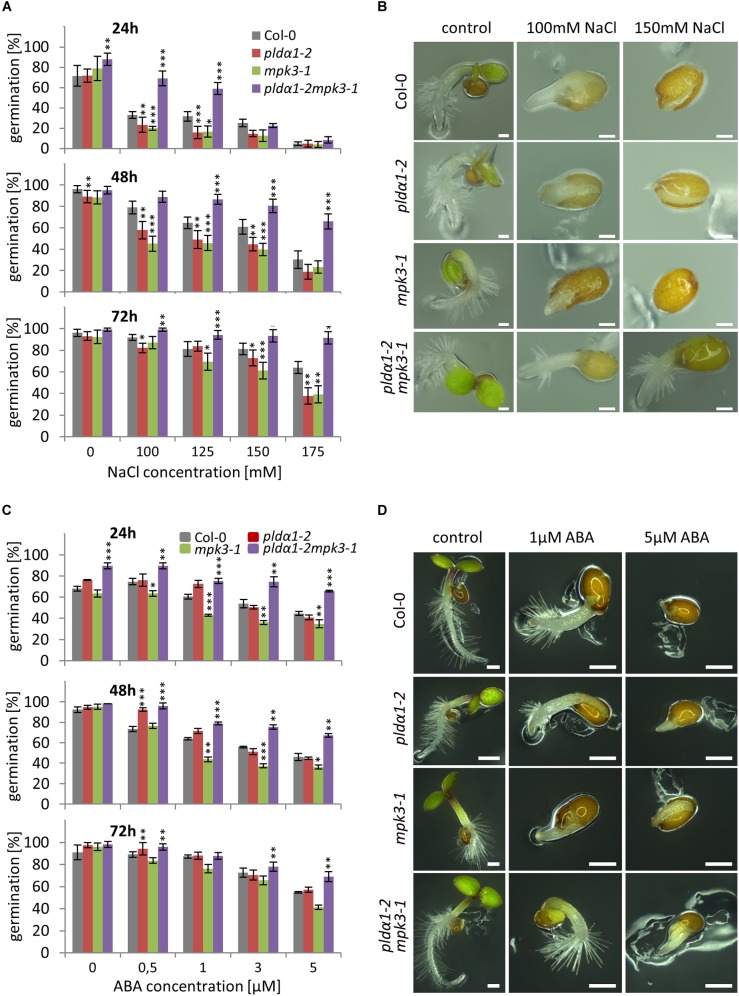
Germination of *PLDα1* and *MPK3* single and double mutant seeds under salt stress and abscisic acid (ABA) treatment. Seeds of wild type (Col-0), *pldα1-2*, *mpk3-1*, and *pldα1-2mpk3-1* were germinated on ½ MS media supplemented with different concentrations of NaCl and ABA, respectively. **(A,C)** Germination rate of seeds under salt stress condition **(A)** and ABA treatment **(C)** were evaluated for radicle emergence at 24, 48, and 72 h. Germination was figured as a percentage of the total number of seeds (*n* = 100). Graphs represent average of three independent experiments. Note that error bars indicate the mean ± SD. Asterisks indicate statistically significant differences in viability of the plants exposed to higher salinity conditions (two-tailed paired *t*-test, ^∗^*p* < 0.05, ^∗∗^*p* < 0.01, ^∗∗∗^*p* < 0.001). **(B,D)** Most abundant germination stages of wild type (Col-0), *pldα1-2*, *mpk3-1*, and *pldα1-2mpk3-1* under salt stress (**B**; 100 and 150 mM NaCl) and ABA treatment (**D**; 1 and 5 μM). Pictures were taken 48 h **(B)** or 72 h **(D)** after transfer of plates to growth chamber. Scale bar = 200 μm.

In order to investigate the genetic interaction between *PLDα1* and *MPK3*, we prepared double mutant plants by crossing *pldα1-2* and *mpk3-1* T-DNA insertion mutants (Supplementary Figure S7). We confirmed the absence of PLDα1 and MPK3 proteins in the above ground parts and roots of homozygous double mutants by immunoblotting analyses using anti-PLD alpha 1/2 and anti-MPK3 antibodies (Figure 5 and Supplementary Figures S8, S9). The analysis also showed the presence of PLDα1 in *mpk3-1* and MPK3 in *pldα1-2* mutant plants, respectively (Figure 5). Images of the entire immunoblots are presented in Supplementary Figures S8, S9. Consistently with published data, seedlings of *pldα1* single mutant showed no obvious phenotypical differences in comparison to the wild type (Bargmann et al., 2009b; Uraji et al., 2012; Zhang et al., 2012; Supplementary Figure S7). Similarly, *pldα1-2mpk3-1* double mutants grown under standard conditions exhibited no obvious morphological phenotypes (Supplementary Figure S7). To shed more light on genetic interaction between *PLDα1* and *MPK3* we have tested the response of *pldα1-2mpk3-1* double mutant to the salt stress and ABA treatment. First, we have tested germination of wild type (Col-0), single *pldα1-2* and *mpk3-1* mutants and *pldα1-2mpk3-1* double mutant exposed to different concentrations of NaCl (0, 100, 125, 150, and 175 mM). Under control conditions, germination ratio of double mutant line was more than 80% after 24 h (Figure 6A,B). Germination of *pldα1-2* and *mpk3-1* single mutants as well as wild type seeds on control media within 24 h was delayed. After 48 h on control media, however, almost all examined seeds germinated (Figure 6A) and reached the same stage of seed germination (Figure 6B). We found that germination rate of double mutant is much higher if compared to both single mutants and wild type. Nevertheless, increasing salt concentration reduced the germination ratio as well as inhibited elongation growth of single mutants and wild type radicles more intensively as compared to the double mutant (Figure 6A,B). Germination ratio of wild type seeds was higher compared to single mutants (Figure 6A,B). These data suggest that seeds of *pldα1-2mpk3-1* double mutant line are more tolerant to salinity in comparison to *pldα1-2* and *mpk3-1* single mutants as well as wild type during germination.

Based on obtained results we next tested effect of ABA treatment on germination ratio of *pldα1-2mpk3-1* double mutant as well as *pldα1-2* and *mpk3-1* single mutant seeds. It is known that accumulation of ABA caused by higher salinity results in germination inhibition (Fujii et al., 2007; Zhu et al., 2007). In our experiments, the highest inhibition of germination was observed with seeds of single mutants and wild type on media containing high ABA concentration (3 and 5 μM) after 48 h (Figure 6C). On the other hand, seeds of *pldα1-2mpk3-1* double mutant showed lowest inhibition of germination in response to ABA. After 72 h, more dramatic inhibition of germination (more than 50%) was observed in the case of both single mutants and wild type on media supplemented with higher concentrations of ABA (3 and 5 μM; Figure 6C,D). The highest tolerance to the high ABA concentration was again observed with seeds of double mutant with only 30% reduction of germination ratio in comparison to the control conditions (Figure 6C,D).

**FIGURE 7:**
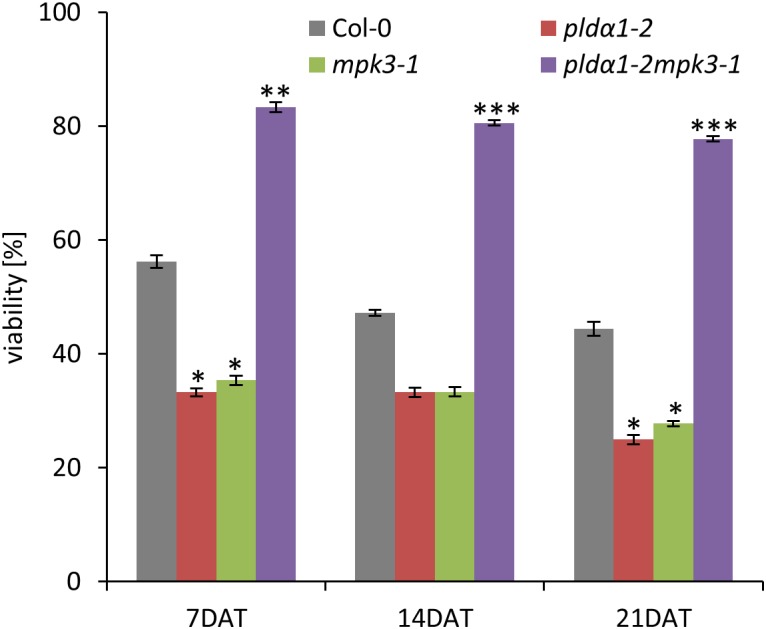
Effect of salt stress on viability of *PLDα1* and *MPK3* single and double mutant plants. Four days old seedlings of wild type, *pldα1-2*, *mpk3-1*, and *pldα1-2mpk3-1* germinated on control media were transferred on media supplemented with 150 mM NaCl. Plant viability was monitored and determined as percentage of survived plants with green true leaves at 7, 14, and 21 days after transfer. Asterisks indicate statistically significant differences in viability of the plants exposed to higher salinity conditions (two-tailed paired *t*-test, ^∗^*p* < 0.05, ^∗∗^*p* < 0.01, ^∗∗∗^*p* < 0.001).

In the second set of experiments we examined survival of double mutant seedlings under salt stress conditions. Four days old seedlings of all plant lines have been grown either on control media or on media supplemented with 150 mM NaCl for next 21 days. Seedlings of *pldα1-2* and *mpk3-1* single mutants were most susceptible to the salt stress leading to the rapid decrease of plant viability observed after 7 DAT as well as 21 DAT (Figure 7). Importantly, double mutant *pldα1-2mpk3-1* showed the best survival ratio during long term cultivation under higher salinity and achieved nearly 80% viability (Figure 7). Thus, the plants of *pldα1-2mpk3-1* double mutant line showed approximately three times higher survival ratio on 150 mM NaCl stress media in comparison to both single mutant lines. These new finding can be connected with the involvement of both proteins in the same signaling pathways.

## Discussion

Abiotic stresses such as high salinity, drought and low temperature determine productivity of crops and are responsible for high crop losses worldwide. It is necessary to better understand plant adaptation processes in order to cope with climate changes and their negative impacts on plants (Munns and Tester, 2008). Such adaptation processes include MAPK and phospholipid signaling networks (Munnik and Testerink, 2009; Šamajová et al., 2013a,b). In this study we found direct connection between PLDα1 and MPK3 and revealed its functional importance in salt stress and ABA responses of Arabidopsis.

There are several ways how PLDα1 might affect salt stress responses of plants. In Arabidopsis PLDα1 produces more PA, which interacts with and stimulates the activity of MPK6 under salt stress conditions. MPK6 in turn phosphorylates plasma membrane Na^+^/H^+^ antiporter (SOS1) which transports Na^+^ ions from cytoplasm to apoplast and maintain ion concentration homeostasis (Yu et al., 2010; Yang and Guo, 2018). This suggests a tight connection between MAPK and PA signaling. Concerning Arabidopsis MAPK pathways participating on salt stress signaling two major cascades were described: MEKK1-MKK2-MPK4/MPK6 and MKK9-MPK3/MPK6, the second one involved also in ethylene signaling (Teige et al., 2004; Xu et al., 2008). According to our results MPK3 could also connect MAPK and PLDα1 signaling.

Binding of the ethylene to ETR1 receptor inactivates constitutive triple response 1 (CTR1), a Raf-like MAPKKK, and thereby activates MKK9-MPK3/MPK6 signaling cascade (Yoo et al., 2008). Moreover, salt stress triggers PA production by PLDα1 and PA binds and inhibits CTR1 and blocks interaction between ETR1 and CTR1 (Testerink et al., 2007). Published data showed that *pldα1* mutant is sensitive to the stress caused by NaCl (Yu et al., 2010) while sensitivity of *mpk3* mutant to NaCl likely depends on the place of T-DNA insertion in the *MPK3* gene (Persak and Pitzschke, 2013; Pitzschke et al., 2014; Zhou et al., 2017). Our results show biochemical and genetic interactions between MPK3 and PLDα1. Interestingly, *pldα1-2mpk3-1* double mutant plants are more resistant to NaCl similarly to *ctr1-1* and *mkk-9* single mutant plants (Yoo et al., 2008; Jiang et al., 2013). We suggest that MPK3, as a downstream target of the CTR1-MKK9 signaling, regulates the response of the Arabidopsis plant to the salt stress by binding and perhaps subsequent phosphorylation of PLDα1. From the previous studies on PA signaling in Arabidopsis it is known that the same impulse (ABA or salicylic acid) can either activate or suppress PA production through PLD or PI-PLC/DGK pathway, respectively (Zhang et al., 2004; Krinke et al., 2009; Kalachova et al., 2016; Pokotylo et al., 2018). We suppose that MPK3 through the binding to PLDα1 can either activate or inhibit its activity. Such evidence was reported in mammalian cells, where protein kinase Cα phosphorylates PLD1 and thus inhibits its activity (Hu and Exton, 2003). Our study provides the first evidence of direct biochemical interaction between MAPK and PLDα1 but to unravel the exact roles of MPK3 and PLDα1 in the salt stress signaling pathway will require more functional studies.

Transient co-expression of PLDα1 with MPK3 showed accumulation and co-localization of these proteins close to the plasma membrane which supports the potential interaction between them. This is in accordance with PLDα1 localization in the vicinity of plasma membrane (Novák et al., 2018). Such co-localization may indicate that MPK3 regulates PLDα1 in order to support its phospholipid hydrolyzing activity. In addition, MPK3 and PLDα1 show common tissue specific expression in root cap cells, trichoblast cells and root hairs as shown by recent study (Novák et al., 2018) and Genevestigator transcriptomic data (Brady et al., 2007). This again shows that these two proteins may commonly contribute to developmental processes ongoing in these tissues.

It is known that *pldα1* mutants are ABA insensitive and they are defective in stomatal movements in response to ABA (Zhang et al., 2004). Salt-induced ABA accumulation inhibits seed germination in plants (Fujii et al., 2007; Zhu et al., 2007) while ABA is capable to trigger PA accumulation (most likely through PLDs) which in turn regulates ABI4 to inhibit germination (Katagiri et al., 2005). It is expected that PLDα1 deficiency would reduce ABA signaling toward germination inhibition of seeds. Nevertheless, *pldα1* mutants showed delayed germination upon salt stress indicating that PLDα1 likely regulates seed germination under salt stress in ABA independent manner. This is emphasized also by the finding that *pldα1* mutants unlike *mpk3* mutant exhibit wild type-like germination rate in response to ABA (Persak and Pitzschke, 2013; Choudhury and Pandey, 2016; our study). In contrast, simultaneous genetic depletion of both *PLDα1* and *MPK3* genes substantially accelerates seed germination under salt conditions. This resembles seed germination of ABA insensitive mutants (Yao et al., 2015). ABA insensitivity of the double mutant was experimentally confirmed in our study. Therefore, we suggest that MPK3 deficiency in absence of PLDα1 hinders the ABA dependent inhibition of seed germination.

Abscisic acid signaling is important for activation of genes involved in salt tolerance (Nakashima and Yamaguchi-Shinozaki, 2013). Simultaneously, such plants can activate the expression of defense genes in ABA independent mechanism, where a *cis*-acting element, termed dehydration-responsive element/cold-responsive element or C-repeat (DRE/CRT), plays an important role (Roychoudhury et al., 2013). From previous research it is known that MAPK cascades play a role in ABA signaling (Danquah et al., 2014). ABA induces transcriptional upregulation of MPK3 and other MAPKs (Wang et al., 2011). Similarly to germination, double mutant plants are more tolerant to salt stress as compared to single mutants and WT. It is likely, that simultaneous inhibition of both genes promotes ABA independent induction of salt stress responsive genes in Arabidopsis.

Previously, MPK3 was shown to phosphorylate lipid transfer protein AZI1 which is able to transfer lipids between membranes *in vitro* (Pitzschke et al., 2014). Here we report PLDα1 as MPK3 interacting protein which modulates salt stress tolerance and ABA response in Arabidopsis.

## Data Availability

All datasets generated for this study are included in the manuscript and/or the Supplementary Files.

## Author Contributions

PV, DN, OŠ, TT, and JC conducted the experiments. OŠ and DN made image post acquisition analyses. PV, OŠ, TT, DN, and JŠ wrote the manuscript with input from all co-authors. JŠ proposed the experiments, supervised this study, participated on data interpretation, and finalized the manuscript.

## Conflict of Interest Statement

The authors declare that the research was conducted in the absence of any commercial or financial relationships that could be construed as a potential conflict of interest.

## Footnotes

1 http://signal.salk.edu/tdnaprimers.2.html
