# Supplementary material for: Biochemical and Genetic Interactions of Phospholipase D Alpha 1 and Mitogen-Activated Protein Kinase 3 Affect Arabidopsis Stress Response
Source: Front Plant Sci. 2019 Mar 18;10:275. doi: 10.3389/fpls.2019.00275 (PMC6431673; doi:10.3389/fpls.2019.00275)
Supplement: Supplementary file 1 [file Data_Sheet_1.PDF]

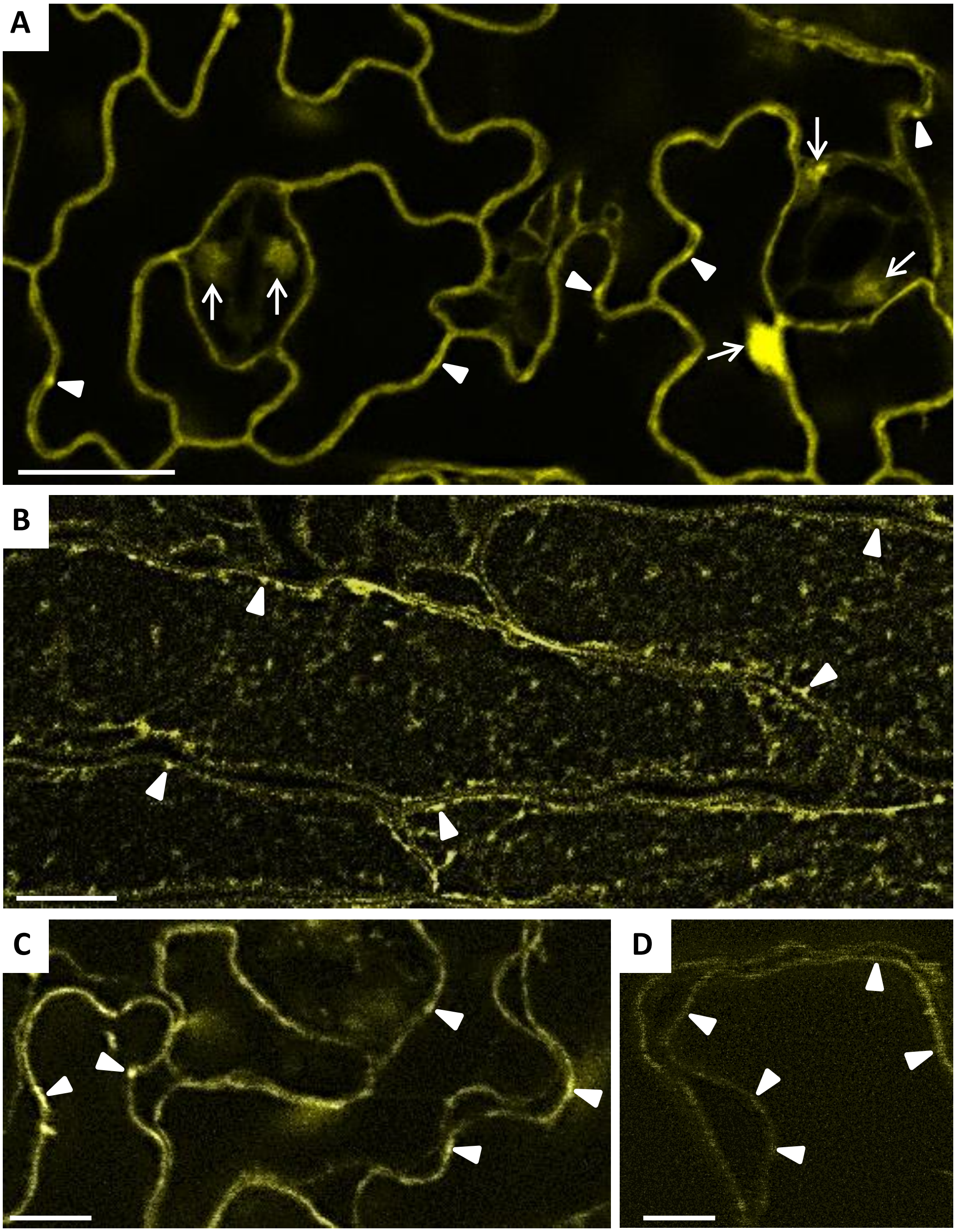

Figure S1. Visualization of YFP-tagged MPK3 expressed under native promoter in living Arabidopsis cells. **(A-D)** YFP-tagged MPK3 is localized to nuclei and nucleoli (arrows in A), to the plasma membrane, especially to distinct PM microdomains (arrowheads in A and B) and to cytoplasmic punctate structures **(B)**. **(C, D)** The PM localization of MPK3-YFP was confirmed by plasmolysis experiment in leaf epidermal cells (arrowheads). Scale bars = 20 µm **(A, B)**, 10 µm **(C, D)**.

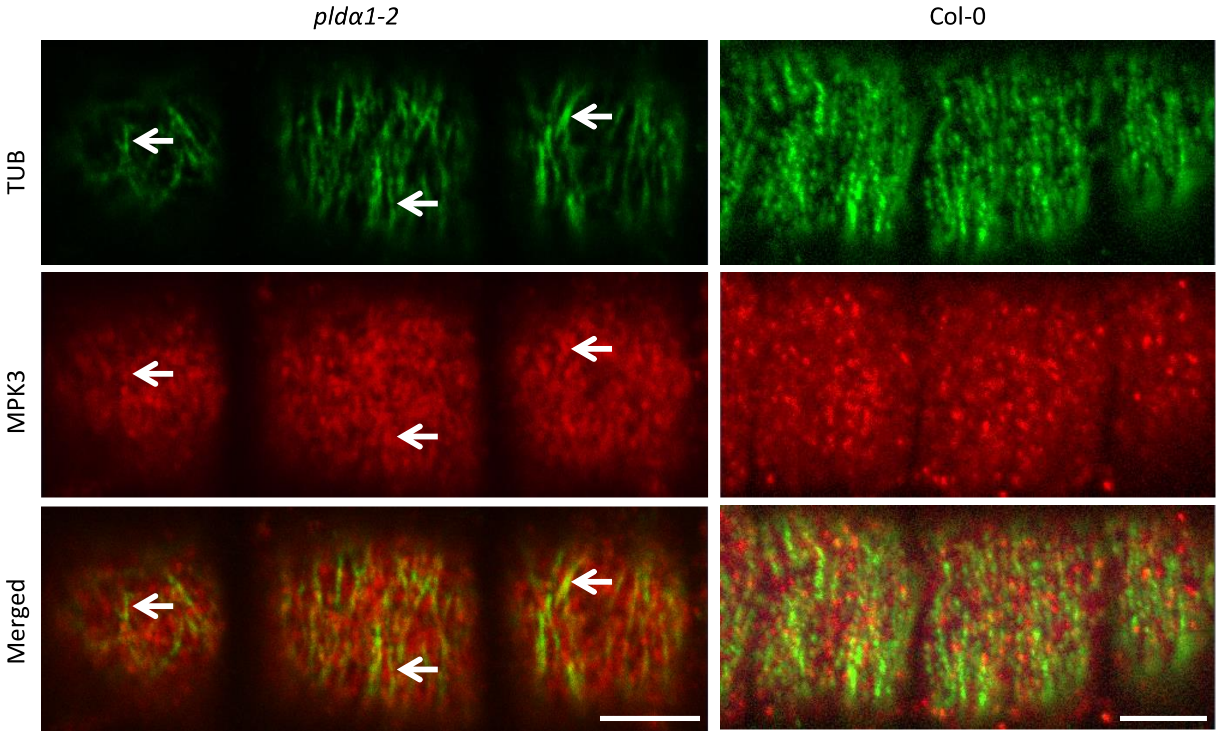

Figure S2. Immunolocalization of cortical microtubules and MPK3 in epidermal root cells of wild type and *pldα1-2* mutant. Scale bar = 5µm.

A

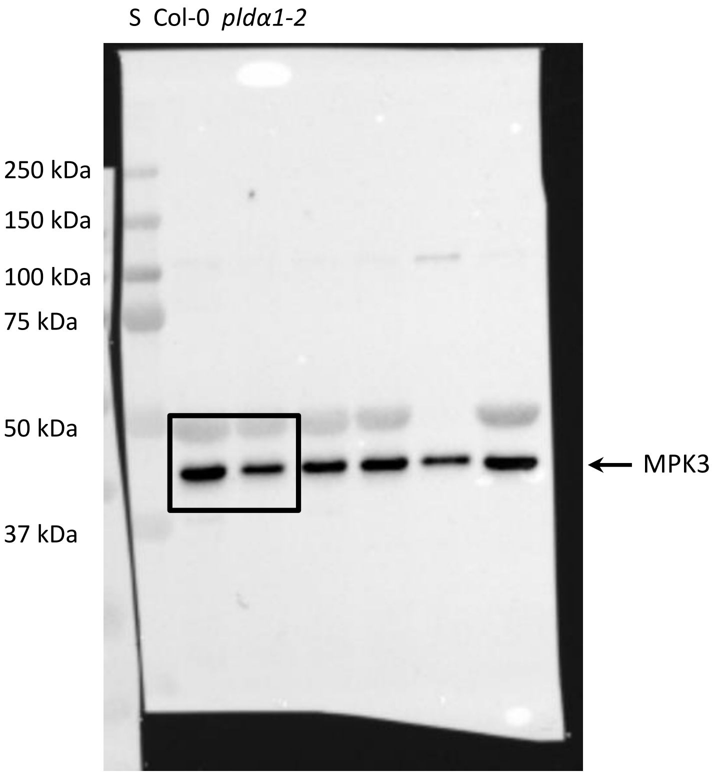

B

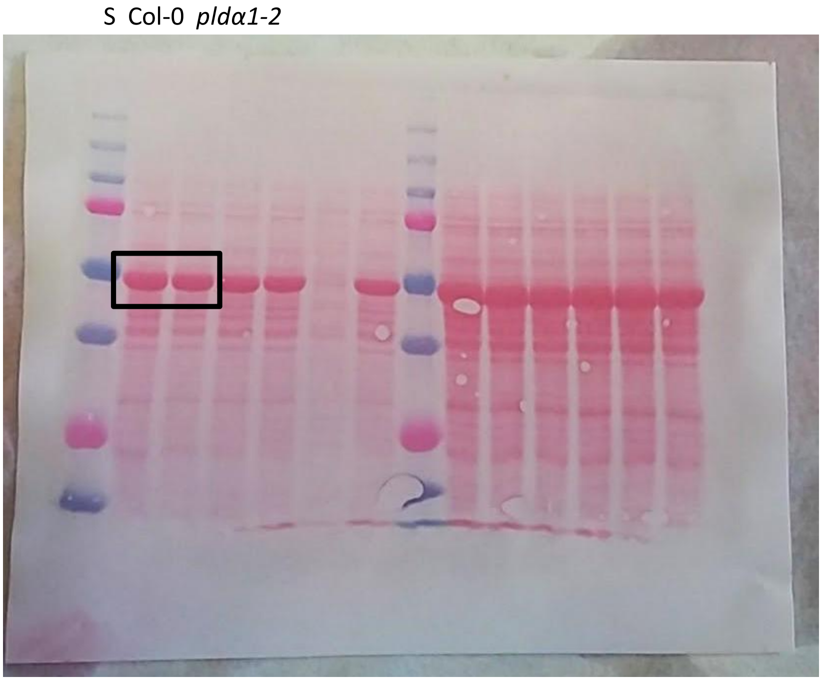

Figure S3. Immunoblotting analysis of MPK3 in *pldα1-2* mutant above ground parts. Full scan of the entire original immunoblot presented in Figure 4A. **(A)** Entire membrane with chemiluminiscent signal observed after probing with anti-MPK3 antibody. The highlighted region shows the section presented in Figure 4A. **(B)** Full image of the respective membrane after staining with Ponceau S. The highlighted region shows the section presented in Figure 4B. Samples loaded on non-annotated lanes are not relevant for this study.

A

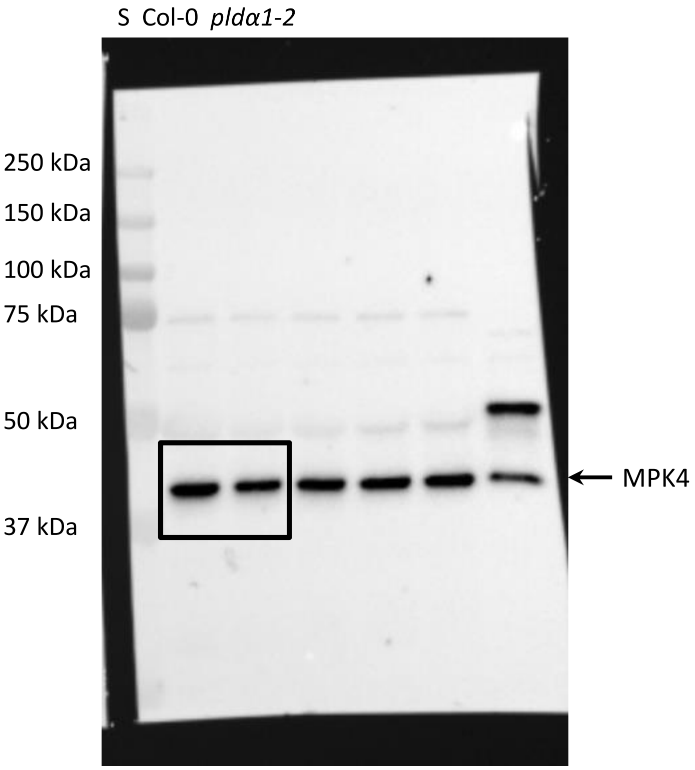

B

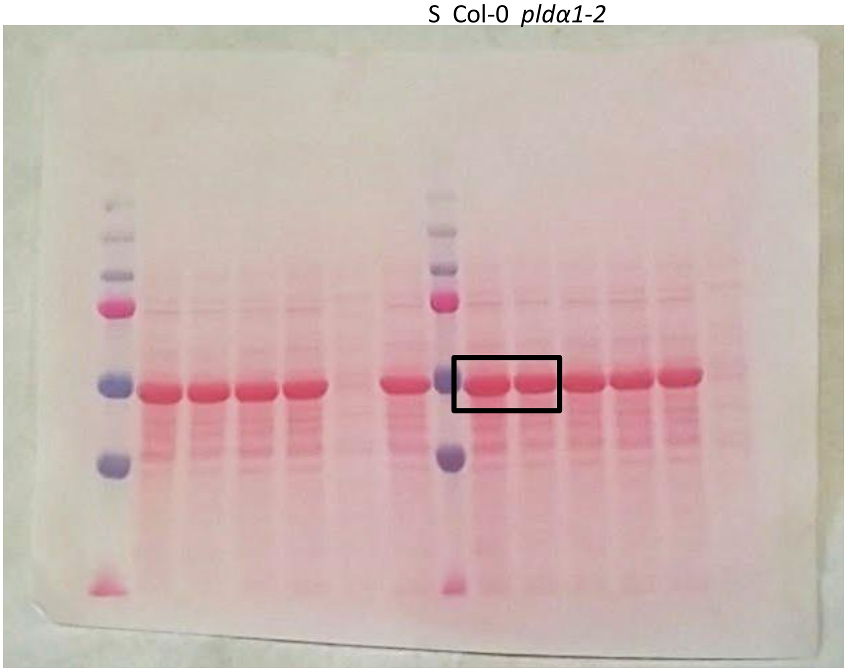

Figure S4. Immunoblotting analysis of MPK4 in *pldα1-2* mutant above ground parts. Full scan of the entire original immunoblot presented in Figure 4A. **(A)** Entire membrane with chemiluminiscent signal observed after probing with anti-MPK4 antibody. The highlighted region shows the section presented in Figure 4A. **(B)** Full image of the respective membrane after staining with Ponceau S. The highlighted region shows the section presented in Figure 4B. Samples loaded on non-annotated lanes are not relevant for this study.

A

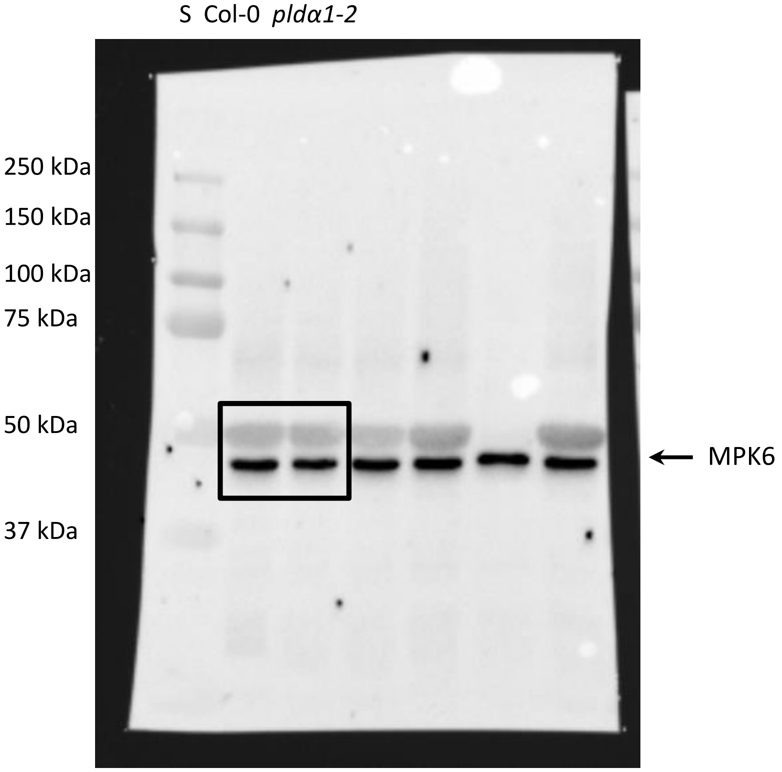

B

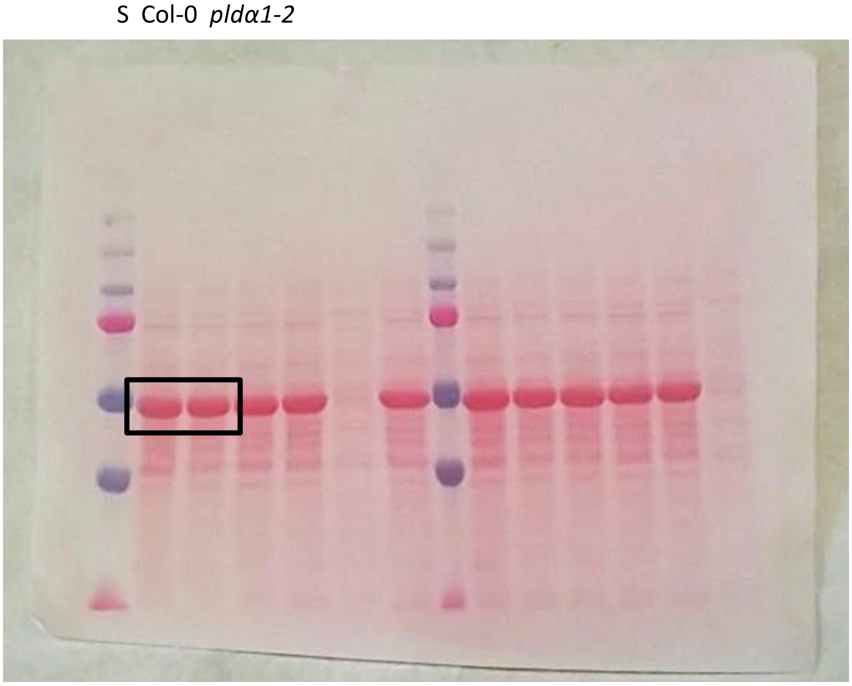

Figure S5. Immunoblotting analysis of MPK6 in *pldα1-2* mutant above ground parts. Full scan of the entire original immunoblot presented in Figure 4A. **(A)** Entire membrane with chemiluminiscent signal observed after probing with anti-MPK6 antibody. The highlighted region shows the section presented in Figure 4A. **(B)** Full image of the respective membrane after staining with Ponceau S. The highlighted region shows the section presented in Figure 4B. Samples loaded on non-annotated lanes are not relevant for this study.

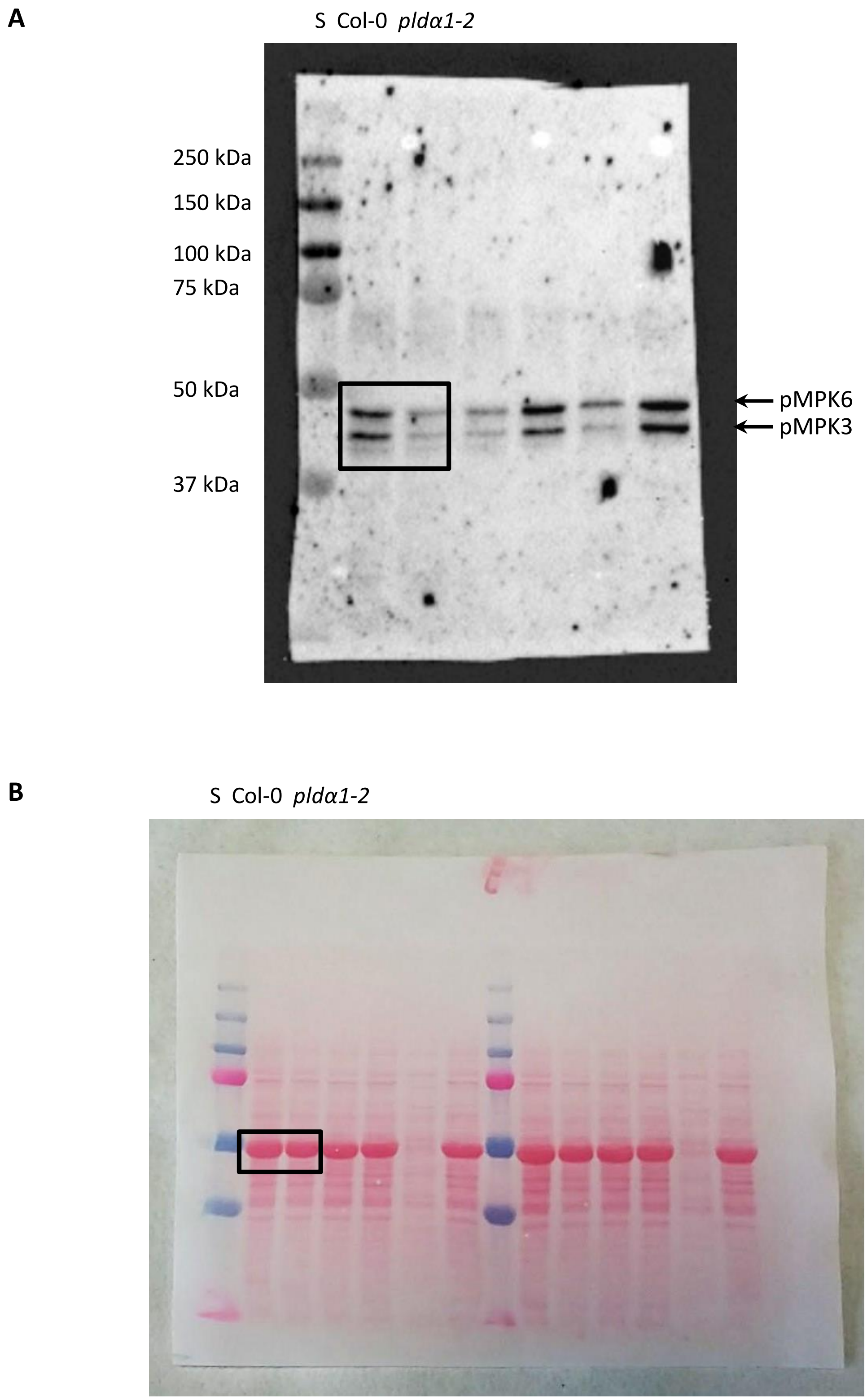

Figure S6. Immunoblotting analysis of phosphorylated MPK3 (pMPK3) and MPK6 (pMPK6) in *pldα1-2* mutant above ground parts. Full scan of the entire original immunoblot presented in Figure 4A. **(A)** Entire membrane with chemiluminiscent signal observed after probing with anti-phospho-p44/42 (pERK) antibody. The highlighted region shows the section presented in Figure 4A. **(B)** Full image of the respective membrane after staining with Ponceau S. The highlighted region shows the section presented in Figure 4B. Samples loaded on non-annotated lanes are not relevant for this study.

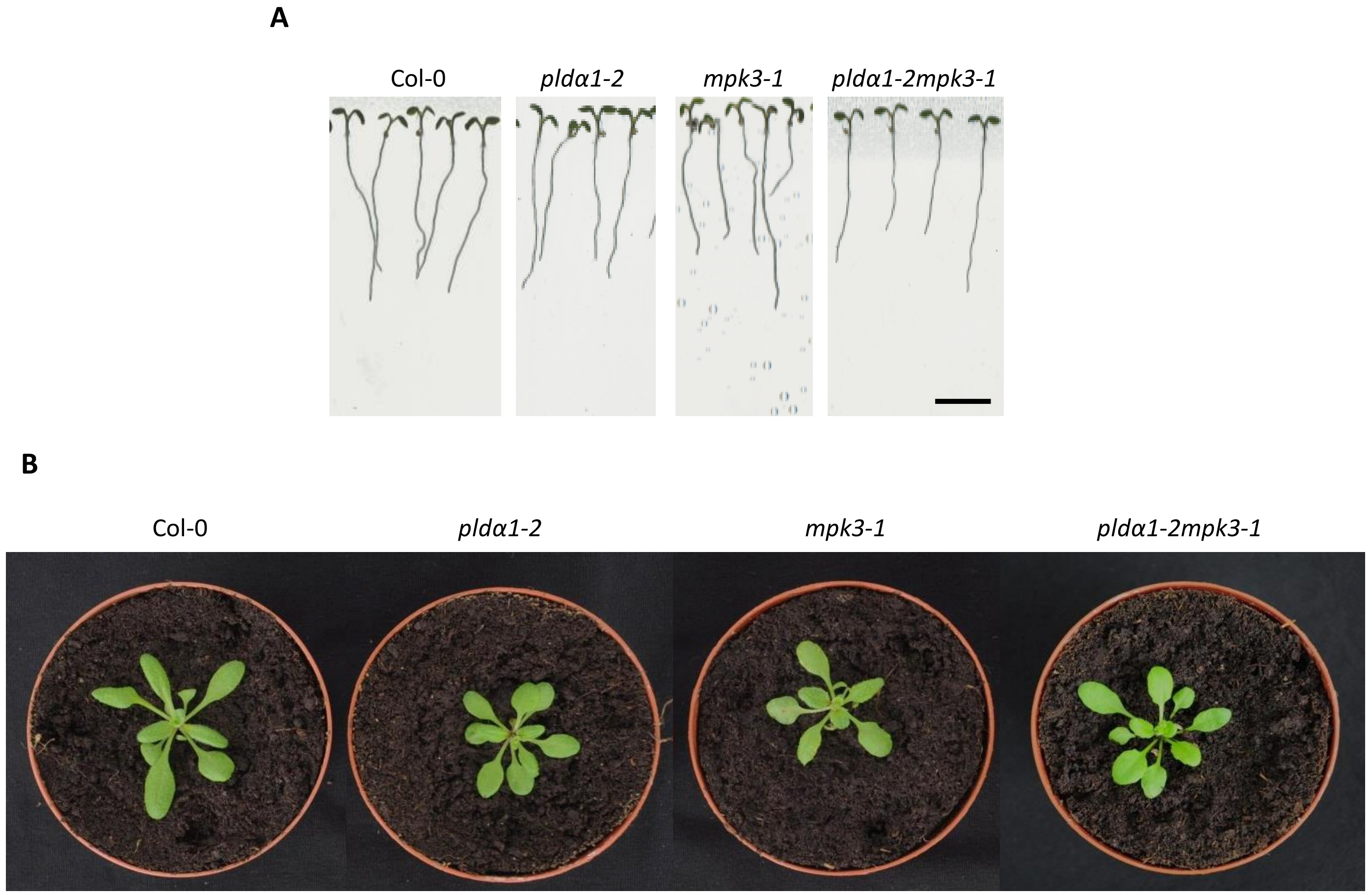

Figure S7. Phenotypic comparison of **(A)** 7 DAG and **(B)** 21 DAG of Col-0 plants, *pldα1-2, mpk3-1* single mutant plants with *pldα1-2mpk3-1* double mutant plants (in F4 generation). Scale bar = 1 cm.

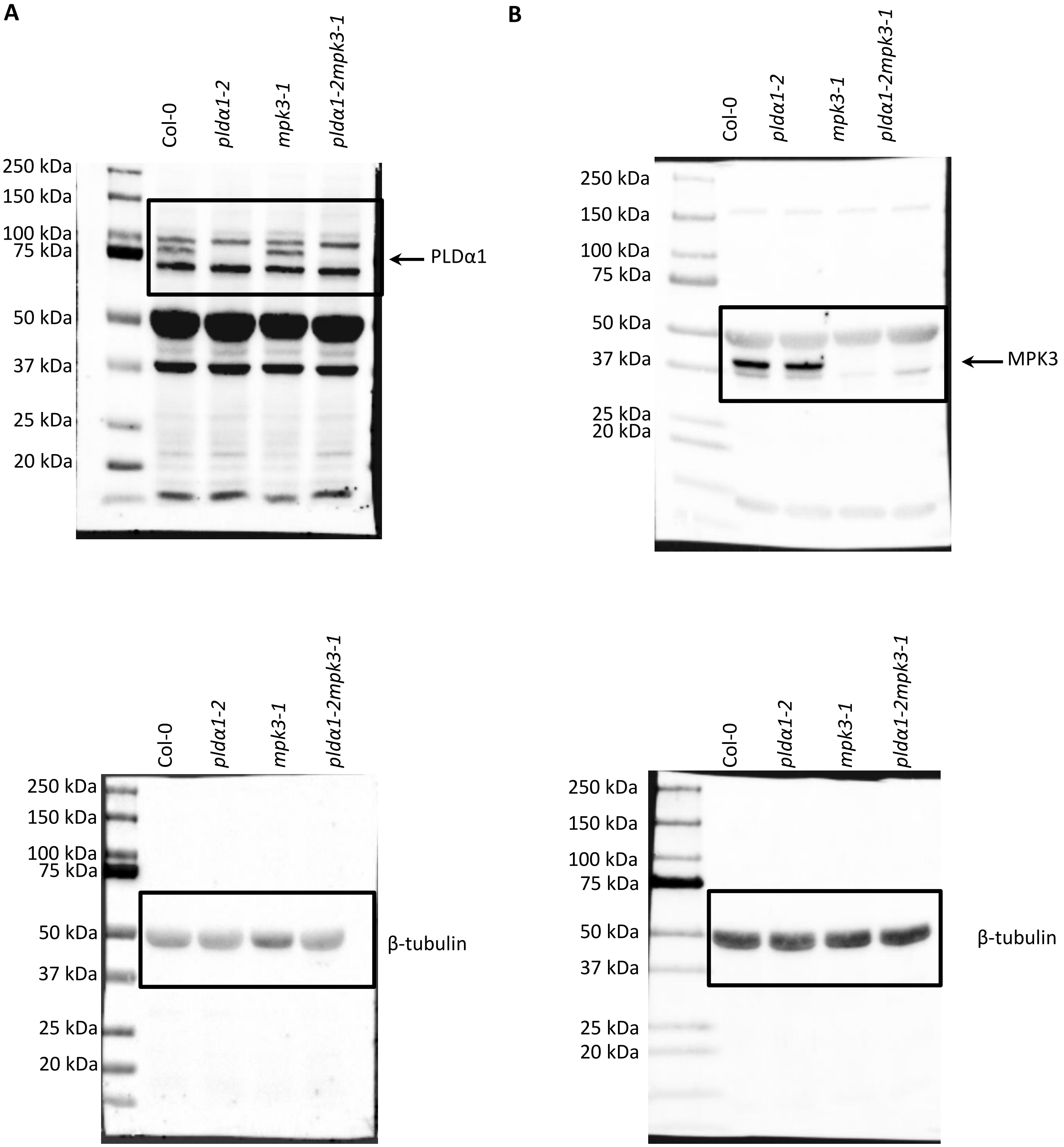

Figure S8. Immunoblotting analysis of the above ground parts of *pldα1-2, mpk3-1* single mutant and *pldα1-2mpk3-1* double mutant plants (in F3 generation). Full scan of the whole immunoblotting membrane probed with with **(A)** anti-PLDα1/2 and **(B)** anti-MPK3 antibodies presented in Figure 5A, B. The highlighted regions showed the sections presented in Figure 5A, B. For loading control the whole immunoblotting membranes were probed with anti-β-tubulin antibody **(A, B)** and the highlighted sections of the membranes are presented in Figure 5A, B.

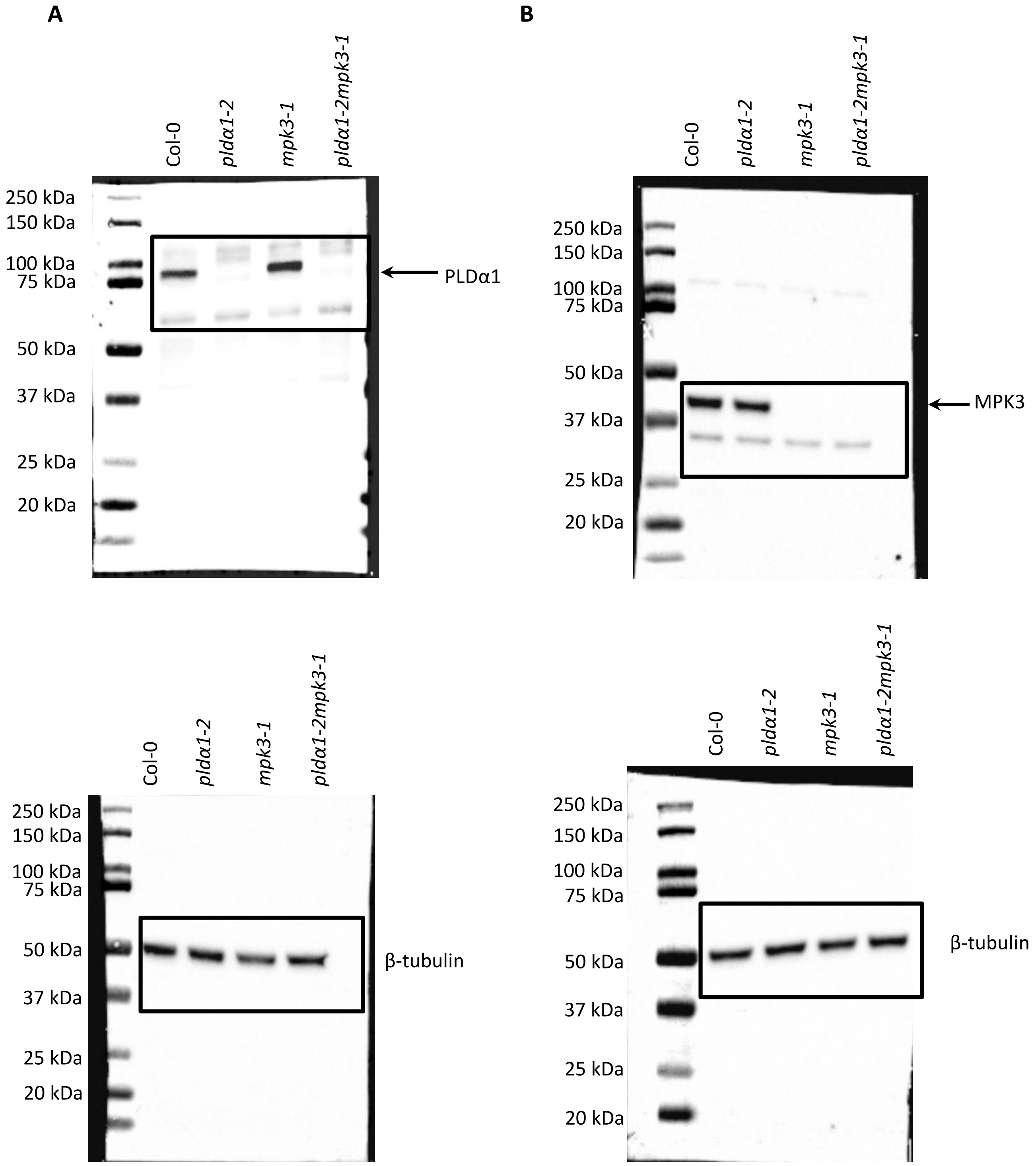

Figure S9. Immunoblotting analysis of the roots of *pldα1-2, mpk3-1* single mutant and *pldα1-2mpk3-1* double mutant plants (in F3 generation). Full scan of the whole immunoblotting membrane probed with with **(A)** anti-PLDα1/2 and **(B)** anti-MPK3 antibodies presented in Figure 5C, D. The highlighted regions showed the sections presented in Figure 5C, D. For loading control the whole immunoblotting membranes were probed with anti-β-tubulin antibody **(A, B)** and the highlighted sections of the membranes are presented in Figure 5C, D.
